# Nobiletin Modulates Adipocyte-Derived Exosomal miRNA to Improve Liver Lipid Metabolism in Obese Mice

**DOI:** 10.1021/acs.jafc.5c06392

**Published:** 2025-08-20

**Authors:** Siu-Yi Leung, Nga-Ying Ho, Chi-Tang Ho, Min-Hsiung Pan

**Affiliations:** † Institute of Food Science and Technology, 33561 National Taiwan University, Taipei 10617, Taiwan; ‡ Department of Food Science, Rutgers University, New Brunswick, New Jersey 08901, United States; § Department of Medical Research, China Medical University Hospital, China Medical University, Taichung City 40402, Taiwan

**Keywords:** obesity, nobiletin, 3T3-L1, adipocyte-derived extracellular vesicles, miRNAs

## Abstract

This study examines the antiobesity properties of nobiletin through its influence on extracellular vesicles, particularly exosomes. Nob-ELVs (nobiletin with adipocyte-derived extracellular vesicles) significantly reduce fat accumulation in adipocytes without compromising cell viability. *In vitro* studies showed that introducing Nob-ELVs to 3T3-L1 preadipocytes led to marked reductions in fat accumulation. RNA sequencing indicated that Nobiletin alters the exosomal miRNA profile, downregulating miR-802-5p and others while upregulating miR-574-5p and related miRNAs. In a high-fat diet mouse model, Nob-ELVs prevented significant weight gain and improved food conversion efficiency. Mice receiving Nob-ELVs exhibited reduced hepatic lipid droplet accumulation and altered protein expressions, enhancing SIRT1 and FGF21 while suppressing ACC, FASN, and CD36. These findings suggest that Nob-ELVs inhibit fat accumulation and enhance lipid metabolism by modulating exosomal miRNAs, offering insights for new obesity treatment strategies.

## Graphical abstract

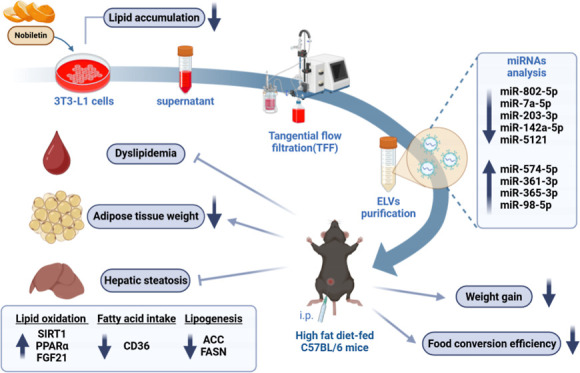

## 1 Introduction

Over the past three decades, changes in dietary patterns and lifestyle habits have contributed to a gradual increase in the prevalence of obesity.^1^ Poor dietary choices and a lack of physical activity result in a higher caloric intake compared to expenditure, which leads to the storage of excess calories as body fat.^2^ Adipose tissue functions as a multifaceted and dynamic endocrine organ that plays a crucial role in regulating systemic homeostasis through the secretion of adipokines, lipids, metabolites, noncoding RNAs, and exosome-like extracellular vesicles (ELVs).^3^ These factors act on metabolic tissues to modulate lipid and glucose homeostasis.^4^ Nonetheless, while healthy adipose tissue produces beneficial systemic effects, the secretion of adipocyte-specific factors is often disrupted in the context of obesity. This dysregulation contributes to the development of low-grade chronic inflammation and insulin resistance.^5^ Adipose tissue is a major source of exosomes and plays a crucial role in intercellular communication.^6^ Therefore, observing the changes in exosomal composition of preadipocytes can provide insights into the physiological state of the cells.

Extracellular vesicles, particularly exosomes, are membrane-bound vesicles of submicron size that are released by cells. These vesicles typically have a diameter ranging from approximately 30 to 150 nanometres.^7^ They are not merely cellular waste products but are crucial mediators of intercellular communication.^8^ Exosomes are derived from multivesicular bodies (MVBs). When MVBs fuse with the plasma membrane, they release intraluminal vesicles into the extracellular environment.^9^ These exosomes are characterized by a high concentration of diverse bioactive molecules, which include proteins, lipids, and nucleic acids, such as mRNA (mRNA), microRNA (miRNA), and other noncoding RNAs.^10^ These molecules carry information from the parent cells and can be taken up by target cells, thereby regulating target cell physiological functions.^11^ These entities serve as functional delivery mechanisms that facilitate communication between cells by transporting their cargo to target cells, thereby impacting the cellular environment.^12^ Exosomes play a significant role in various physiological and pathological processes, including cell proliferation, differentiation, immune responses, metabolic regulation, and tumorigenesis.^13^ They not only transmit signals between cells but also have potential as diagnostic and therapeutic targets for diseases.^14^ Adipocytes possess the ability to differentiate from preadipocytes, and the 3T3-L1 preadipocyte cell line is commonly utilized to investigate the mechanisms underlying adipocyte differentiation. Furthermore, this cell line serves as an *in vitro* model for adipocytes, enabling research into insulin signaling pathways, obesity, and cardiovascular diseases.^15^ The differentiation of adipocytes, initiated by 3T3-L1 preadipocytes in response to inducers such as insulin, 3-isobutyl-1-methylxanthine (IBMX), and dexamethasone (DEX), may lead to modifications in the contents of extracellular vesicles released by these cells. This phenomenon can be attributed to alterations in the physiological state of the parent cells, which may subsequently influence metabolic regulation.

Regarding their association with obesity, exosomes play complex roles. Adipose tissue releases exosomes that can influence systemic metabolic balance.^16^ Studies have shown that the number and content of exosomes released by adipocytes in obese individuals differ from those in nonobese individuals.^17,18^ These exosomes may carry pro-inflammatory molecules, leading to obesity-related pathologies such as insulin resistance and chronic inflammation.^19^ Furthermore, exosomes derived from adipocytes can transfer lipids to macrophages, affecting macrophage function.^20^ In obese animal models, a high-fat diet increases the amount of exosomes released by adipose tissue.^18^ In contrast, specific stimuli associated with a reduction in body weight, such as starvation and rapamycin, significantly reduce exosome secretion.^21^ The microRNAs within these exosomes can regulate gene expression in other tissues, such as the liver, thereby affecting metabolism.^22^ Additionally, hypoxic adipocytes release exosomes enriched in lipogenic enzymes, promoting lipid accumulation in neighboring cells.^23^ Studies have found changes in the levels of circulating exosomal miRNA in the blood of obese individuals, which may affect insulin sensitivity.^24^ Exosomes from macrophages can also impact insulin resistance.^25^ In addition, endothelial cells may communicate with adipocytes through exosomes, thereby affecting the metabolism of adipose tissue.^26^ Furthermore, research has indicated that exosomes isolated from the blood of mice fed a high-fat diet, when repeatedly injected into mice on a normal diet, can lead to the accumulation of immature bone marrow cells CD11b­(+)­Ly6C­(hi)­Ly6G(−) in the liver. This accumulation results in chronic inflammation and promotes obesity-related diseases, such as nonalcoholic fatty liver disease.^27^ Overall, exosomes play a multifaceted role in the pathophysiology of obesity, both contributing to obesity-related inflammation and metabolic disorders and serving as potential therapeutic targets.

Among the most common polymethoxyflavones (PMFs) in citrus peels are tangeretin, and nobiletin.^28^ Nobiletin is a bioactive PMF known for its antioxidant,^29^ anti-inflammatory,^30^ antihypertensive,^31^ and neuroprotective properties.^32^ Nobiletin has the potential to modulate lipid metabolism^33^ and research indicates that it can affect adipocyte function through various mechanisms.^34^ For example, nobiletin can reduce lipid accumulation in adipocytes^35^ and regulate the expression of genes related to adipogenesis.^28^ Additionally, it can decrease lipid accumulation and pro-inflammatory cytokine secretion in macrophages by regulating the expression of miR-590 .^36^ Nonetheless, the limited crystallinity, elevated melting point, inadequate water solubility, and low oral bioavailability of nobiletin significantly hinder its application in food systems.^37^ Exosomes, owing to their distinct properties as a proficient drug delivery system, have significantly revolutionized the pharmaceutical landscape. Additionally, several studies indicate that exosomes may function as a vehicle for encapsulating unstable drug compounds, such as curcumin, thereby potentially augmenting their therapeutic efficacy.^38^ The application of exogenous stimuli to host cells has been demonstrated to effectively augment the yield of exosomes in biomanufacturing processes. This approach has been validated as a feasible strategy for enhancing exosome production while preserving the fundamental properties of the cell membrane.^39^

This research endeavor seeks to enhance the comprehension of exosomes, with a particular focus on the role of nobiletin in mitigating lipid accumulation within the 3T3-L1 adipocyte *in vitro* model. The study further aims to extend these findings to an *in vivo* model utilizing obese mice, thereby addressing the challenges associated with the low absorption rate and bioavailability of nobiletin. Specifically, the investigation examines the effects of exosomes treated with nobiletin on lipid deposition in both cellular and animal models, with the objective of elucidating their influence on fat lipid accumulation and related metabolic disorders. A central hypothesis posited in this study is that exosomes secreted by nobiletin-stimulated 3T3-L1 adipocytes modulate hepatic lipid metabolism via miRNA signaling pathways. Through mechanistic analyses, the research reveals that Nob-ELVs can counteract the significant elevation of miR-802-5p induced by ob-ELV. Furthermore, Nob-ELVs is shown to enhance the expression of proteins associated with liver lipid oxidation while concurrently diminishing the levels of proteins linked to lipid synthesis. These findings contribute novel insights into the metabolic alterations associated with obesity and present potential therapeutic targets for addressing lipid metabolic disorders linked to nobiletin-treated exosomes.

## 2 Materials and Methods

### 2.1 Materials

Nobiletin (98% purity) was purchased from Nanjing Spring & Autumn Biological Engineering (Nanjing, China).

### 2.2 Cell Culture and Differentiation of Adipocytes

Mouse 3T3-L1 preadipocytes (American Type Culture Collection) were cultured in DMEM supplemented with 2 mM glutamine, 1% penicillin/streptomycin, and 10% fetal calf serum at 37 °C with 5% CO_2_. After 8 days of differentiation in DMI (insulin, IBMX, DEX, and rosiglitazone), the cells were seeded into a 24-well plate (2.5 × 10^4^ cells/mL) or a 10 cm dish. Once confluent, cells were incubated in DMEM with 10% FBS and 5 μg/mL insulin, 0.5 mM IBMX, 1 μM DEX, and 2 μM rosiglitazone for 48 h. The medium was then changed to DMEM with 10% FBS and 5 μg/mL insulin for an additional 2 days. From day 4 onward, cells were maintained in DMEM with 10% FBS, with medium changes every 2 days until day 8, forming the DMI­(+) group. Varying concentrations of nobiletin were introduced from day 0 to day 8. The DMI(−) group received no insulin, IBMX, DEX, or rosiglitazone.

### 2.3 Cell Viability

3T3-L1 preadipocytes (2.5 × 10^4^ cells/mL) were cultured in DMEM supplemented with 10% FBS overnight in 96-well plates. Subsequently, the cells were treated with nobiletin at concentrations of 60, 80, 100, or 120 μg/mL for 24 h. Following treatment, 20 μL of MTT solution were introduced into each well, and the cells were incubated at 37 °C for an additional 3 h. The MTT-containing media was then carefully aspirated, and 100 μL of dimethyl sulfoxide (DMSO) was added to solubilize the intracellular formazans. The resulting formazan levels were quantified using an ELISA, with absorbance measured at a wavelength of 570 nm.

### 2.4 Oil Red O Staining

The lipid accumulation in 3T3-L1 cells on day 8 was assessed using Oil Red O (ORO) staining. Initially, the cells were subjected to two washes with PBS and subsequently fixed in 10% formalin at 4 °C for one night. The ORO stock solution was prepared by diluting it with isopropanol to a concentration of 5 mg/mL, followed by filtration through a 0.22 μm membrane filter of mixed cellulose esters (MCE). The cells were then stained with the ORO solution, which had been diluted with distilled water, for a period of 5 min at room temperature. After three additional washes with PBS, the lipids within the cells were extracted using isopropanol, and the absorbance was measured at a wavelength of 510 nm to quantify the lipid content.

### 2.5 Isolation of Adipocyte-Derived Extracellular Vesicles

3T3-L1 cells were cultured and treated with DMI and 120 μM nobiletin. The culture medium was collected to extract ELVs, designated as Nob-ELVs. Untreated preadipocyte ELVs were referred to as pre-ELVs, while ELVs from differentiated mature adipocytes were labeled as ob-ELVs. Postcollection, a tangential flow filtration (TFF) system was used to further concentrate and filter the media. ELVs were isolated using a Lefoscience MAP.03-plus tangential flow filtration system. A total of 450 mL of culture medium was filtered through hollow fiber membranes (750 kDa cutoff) to remove cellular debris. Filters were washed with sterile PBS and treated with 0.5 N sodium hydroxide to eliminate contaminants. Biological samples were processed at a flow rate of 90 mL/min, concentrating the ELVs solution to 10–12 mL, which was aliquoted and stored at −80 °C until analysis.

### 2.6 Nanoparticle Tracking Analysis (NTA)

The quantitative characterization of the size distribution, mean, mode, and median central tendency, along with the particle concentration of ELVs, was conducted using NTA. ELVs isolated from adipocytes were diluted in PBS to meet the appropriate concentration (1000 μL PBS and 100 μL ELVs) and were analyzed on NanoSight NS300 (Malvern Panalytical, UK) in National Taiwan University Hospital.

### 2.7 Transmission Electron Microscopy (TEM)

TEM was employed to assess the morphology of exosomes derived from adipocytes. The exosomes were deposited onto holey carbon-coated TEM grids, which were subsequently dried for 10 min in the dark. Excess liquid samples were eliminated using dry filter paper, followed by fixation with a 2% uranyl acetate solution. After 1 min, any surplus uranyl acetate was removed with dry filter paper, and the grids were allowed to dry overnight within the grid box. The exosomes were then visualized using a transmission electron microscope operating at an acceleration voltage of 120 kV (JEM-1400, JEOL, Tokyo, Japan).

### 2.8 Exosomes Uptake Assay

Adipocytes were cultured at a density of 2.5 × 10^4^ cells per well in a 24-well plate for 24 h before the uptake experiment. The cells were washed with sterile PBS to remove the culture medium and residual extracellular vesicles, then labeled with DAPI (200× dilution) for 30 min in the dark. After washing off unbound DAPI, cells were examined under fluorescence microscopy to visualize stained DNA. Adipocyte-derived extracellular vesicles were labeled using the PKH67 Fluorescent Cell Linker Kit (Sigma-Aldrich). PKH67 dye was mixed with extracellular vesicles and subjected to ultrafiltration at 3000*g* for 10 min at 4 °C. The supernatant containing labeled extracellular vesicles was collected, incubated at room temperature for 30 min, and added to the adipocytes. After 12 h at 37 °C, cells were washed with PBS and observed under a microscope.

### 2.9 Purification of Total RNA

Total RNA was extracted from adipocyte-derived extracellular vesicles using the miRNeasy Mini Kit (QIAGEN-217004). The process involved lysing the extracellular vesicles with QIAzol Lysis Reagent, adding chloroform for phase separation, and centrifuging to isolate the aqueous phase. The RNA was then mixed with ethanol, applied to the miRNeasy Mini Spin Column, and washed with Buffer RWT and Buffer RPE. Finally, RNase-free water was used to elute the purified RNA.

### 2.10 Extracellular Vesicles miRNA Sequencing

Total RNA samples stored at −80 °C were sent to Genomics, BioSci & Tech Co. Ltd. for analysis. The QIAseq miRNA Library Kit was used to construct the sequencing library, with adapters ligated to small RNA molecules. First strand cDNA synthesis was performed using the QIAseq miRNA NGS RT enzyme. Following PCR amplification, size selection was conducted for fragments of 170 to 200 base pairs using QIAseq magnetic beads. The library’s quality was assessed with the Qsep400 system and Qubit 2.0 fluorometer. Libraries meeting quality standards were sequenced on the Illumina NovaSeq 6000 platform, producing trimmed 75 bp single-end reads. Adapter sequences were removed using Trim Galore, and reads were aligned to the reference genome and miRBase v.22.1 with Bowtie. Postalignment, BAM files were processed with SAMtools, and miRNA expression profiles were normalized using edgeR. Differentially expressed miRNAs (DEmiRNAs) were identified via DEGSeq, with target predictions performed using miRanda, TargetScan, and PITA.

### 2.11 Experimental Animals

Four-week-old male C57BL/6 mice were procured from the National Laboratory Animal Center in Taipei, Taiwan. The mice were maintained in an environment at a controlled temperature of 24 ± 2 °C and 55% relative humidity, under a 12 h light/dark cycle, with unrestricted access to food and water. After a one-week acclimation, mice were randomly assigned to five groups: ND (normal diet, 15% fat, saline injections), HFD (high-fat diet, 60% fat, saline injections), HFD + pre-ELVs, HFD + ob-ELVs, and HFD + Nob-ELVs. Mice were injected intravenously with ELVs (30 μg/mouse).^40^ Each group included six mice, with treatments administered every 3 days for 10 weeks. Food consumption was assessed daily and body weight was recorded on a weekly basis over a period. The following equation was used to calculate the food efficiency ratio (FER): FER (%) = total body weight gain (g)/total food intake (g) × 100. Upon completion of the study, all subjects underwent an overnight fasting period before sacrifice via CO_2_ anesthesia. Blood samples were obtained from the cardiac region for subsequent biochemical analysis. Additionally, the liver, spleen, kidneys, and intra-abdominal adipose tissue were excised and weighed. The collected tissue samples were preserved at −80 °C prior to analysis. All the procedures were approved by the Institutional Animal Care and Use Committee of National Taiwan University (NTU-111-EL-00143, IACUC, NTU).

### 2.12 Biochemical Analysis

Plasma samples were subjected to centrifugation at 3500*g* for 10 min at a temperature of 4 °C and the separated serum was stored at −80 °C for future analysis. The serum samples were sent to the National Laboratory Animal Center (Taipei, Taiwan) for the assessment of various biochemical markers, including aspartate aminotransferase (AST), alanine aminotransferase (ALT), triglycerides (TG), total cholesterol (TC), high-density lipoprotein cholesterol (HDL-C), and low-density lipoprotein cholesterol (LDL-C).

### 2.13 Histological Analysis of Liver

Liver was fixed in 10% formalin for 24 h and then dehydrated with a sequence of ethanol solutions and embedded in paraffin. Tissue sections (5–6 μm) were cut and stained with hematoxylin and eosin (H&E). Images were taken using an Olympus microscope under 200× magnification.

### 2.14 Western Blotting

Liver tissues were weighed, homogenized, and lysed in cold gold lysis buffer with a protease inhibitor for 1 h on ice. After centrifugation at 12,000 rpm for 30 min at 4 °C, the supernatant was collected. Adipocyte-derived extracellular vesicles were lysed in RIPA buffer, incubated on ice for 1 h, and centrifuged at 15,000 rpm for 10 min to collect the supernatant for analysis. Protein concentration was measured using the Bradford method. Equal amounts of protein were mixed with dye and heated at 100 °C for 10 min before separation by 10–15% SDS-PAGE. Proteins were transferred to PVDF membranes and blocked with a solution for 1 h at room temperature. Membranes were incubated overnight at 4 °C with primary antibodies, then washed with TPBS. After incubation with HRP-conjugated secondary antibodies, blots were developed using enhanced chemiluminescence (ECL).

### 2.15 Statistical Analysis

Experimental data were analyzed using the statistical software GraphPad Prism 9. Results are presented as mean ± standard deviation (mean ± SEM). When the experimental results contained only one independent variable, differences between groups were examined using one-way analysis of variance (one-way ANOVA), and Tukey’s method was used to test for differences between sample groups. A *p*-value of <0.05 was considered to indicate a significant difference between groups.

## 3 Results

### 3.1 Nobiletin Inhibits Lipid Accumulation in 3T3-L1 Cells

In the Oil Red O staining experiment, the assessment of oil droplet morphology via microscopy and the quantification of absorbance values facilitated the identification of crops with the potential to inhibit adipocyte formation. The results indicate that, compared to the DMI(−) group, adipocytes in the DMI­(+) group were stained red, demonstrating the successful induction of preadipocyte differentiation into mature adipocytes with lipid accumulation. The intensity of the Oil Red O staining in the sample groups decreased with increasing concentrations of nobiletin (Figure 1A). Quantitative analysis revealed that concentrations of 60 and 80 μM nobiletin did not effectively inhibit lipogenesis, whereas concentrations of 100 or 120 μM were required to significantly reduce lipid droplet formation (Figure 1B). Furthermore, the cytotoxicity of varying concentrations of nobiletin on 3T3-L1 cells was evaluated using the MTT cell viability assay, which confirmed that none of the four concentrations of nobiletin resulted in decreased cell viability. This indicates that the observed effects of 100 or 120 μM nobiletin in reducing lipid accumulation in adipocytes were not attributable to cell death (Figure 1C). In subsequent experiments, 3T3-L1 cells will be cultured with nobiletin at a concentration of 120 μM (Figure 1D). Following the collection of the cell culture medium, TFF will be employed. Throughout the filtration process, the permeate that traverses the membrane will be collected in a designated container, while the residual liquid will be recirculated for refeeding. This approach aims to enhance the concentration of exosomes secreted by the preadipocytes, which will subsequently be assessed for their antiobesity efficacy (Figure 1E).

**Figure 1:**
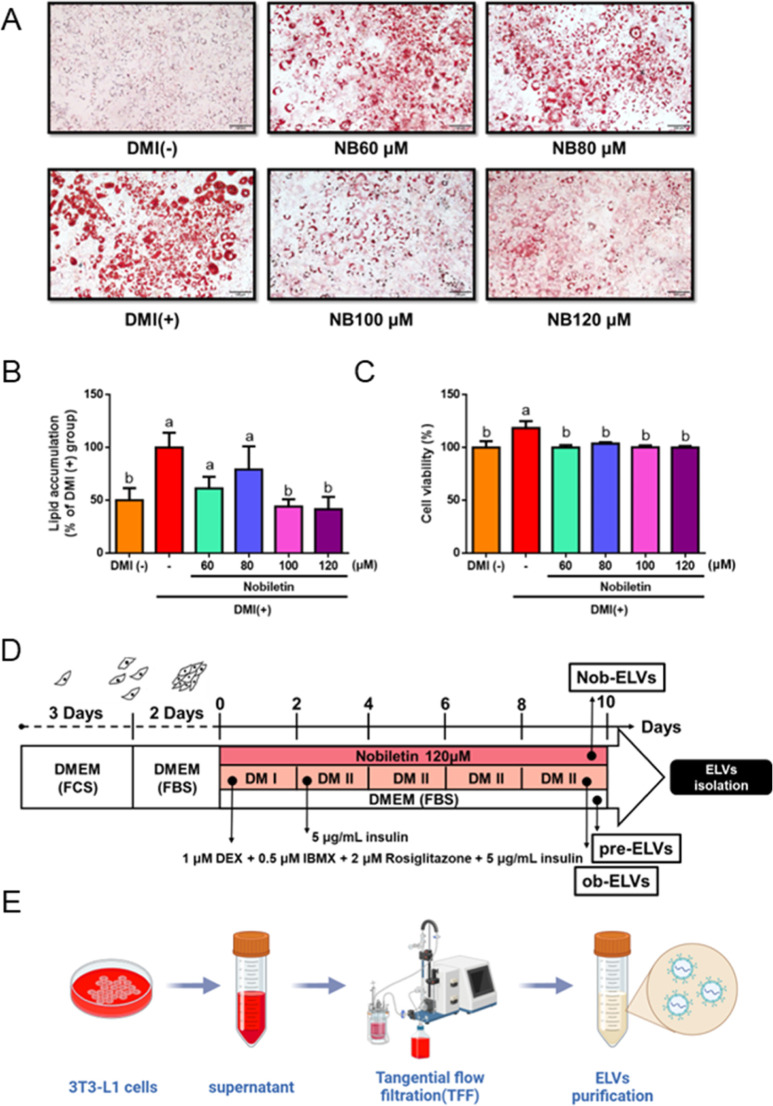
Nobiletin inhibits lipid accumulation in 3T3-L1 cells. (A) Representative photomicrographs of Oil Red O (ORO) stained in 3T3-L1 adipocytes. (B) Effect of nobiletin on lipid accumulation in 3T3-L1 adipocytes, determined using ORO staining. (C) Effect of nobiletin on cell viability in 3T3-L1 adipocytes, determined using a MTT assay. (D) Diagram of adipocyte culture for the three groups of 3T3-L1 adipocytes with or without the addition of DMI and with the addition of nobiletin. (E) The TFF system was employed to isolate ELVs. Data are expressed as mean ± SEM (*n* = 3). Values with different letters are significantly different, *p* < 0.05.

### 3.2 Characterization of Adipocyte-Derived Extracellular Vesicles

The existing literature indicates that current methodologies for identifying exosomes predominantly emphasize their structural characteristics, size, and the surface proteins incorporated during the exosome formation process.^41^ NTA conducted on exosomes released by preadipocytes revealed the following size distribution of extracellular vesicles: pre-ELVs measured 120.5 ± 1.2 nm, ob-ELVs measured 109.3 ± 3.5 nm, and Nob-ELVs measured 113.4 ± 2.1 nm. The concentrations of these extracellular vesicles were quantified as follows: pre-ELVs at 1.43 × 10^12^ ± 7.89 × 10^10^ particles/ml, ob-ELVs at 8.35 × 10^10^ ± 4.52 × 10^9^ particles/ml, and Nob-ELVs at 6.49 × 10^10^ ± 3.52 × 10^9^ particles/ml (Figure 2A).

**Figure 2:**
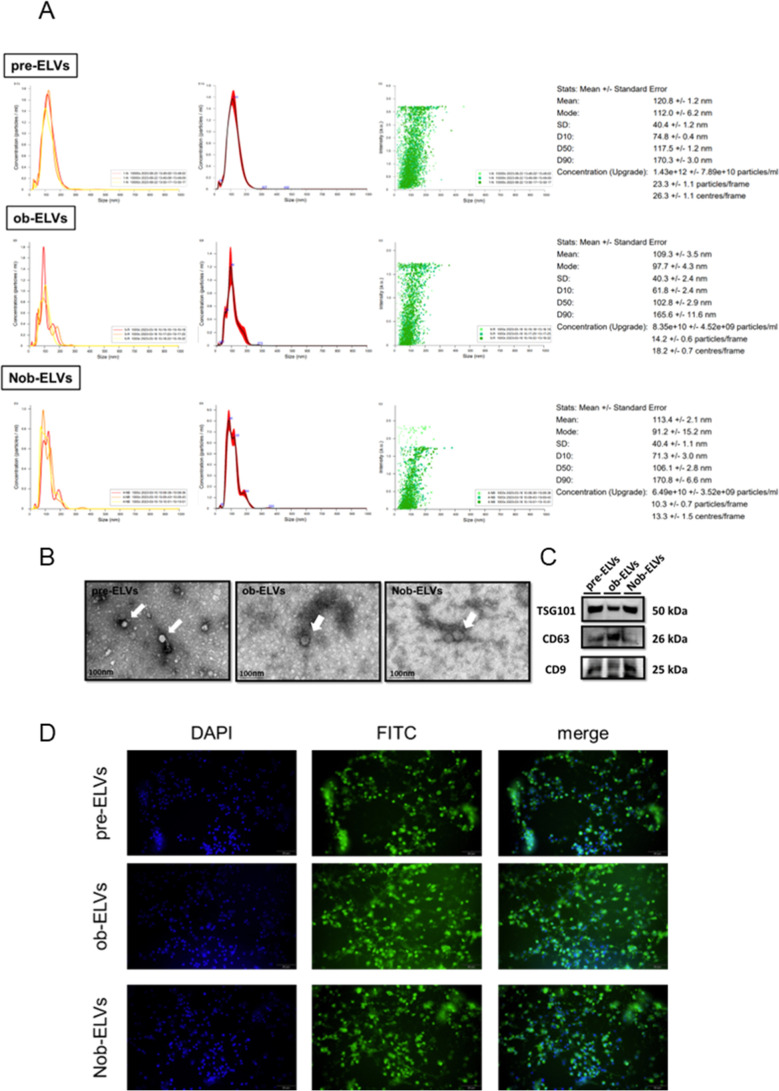
Characterization of pre-ELVs, ob-ELVs and Nob-ELVs. (A) Particle size distribution of exosomes detected by NTA. (B) Representative TEM of exosomes. Scale bars 100 nm. (C) Western blotting analysis of the expression of exosomal protein biomarkers TSG101, CD63, and CD9. (D) Merged images showing quantification of internalized PKH26-labeled adipocyte-derived extracellular vesicles counterstained with Hoechst (blue). Samples per group, *n* = 3. Scale bar 50 μm (micrograph).

To further validate the structural integrity of the exosomes, negatively stained samples were examined using electron microscopy. This analysis identified typical secretory extracellular vesicles within the isolated fractions, demonstrating that the exosomes exhibited a disc-like morphology, with an estimated diameter ranging from approximately 40 to 100 nm. The structural and dimensional characteristics of the three groups of ELVs align with established literature, which describes exosomes as having sizes between 40 and 160 nm (Figure 2B). Exosomes are characterized by a variety of specifically expressed proteins, including CD63, CD9, and TSG101. Recent studies have leveraged this property for the preliminary identification of isolated exosomes.^42^ Western blot analysis confirmed the expression of CD63, CD9, and TSG101 proteins in pre-ELVs, ob-ELVs, and Nob-ELVs (Figure 2C), thereby substantiating the successful extraction of extracellular vesicles secreted by adipocytes for further investigation. As a signaling carrier, the ability of extracellular vesicles to effectively enter adipocytes is crucial for exploring their mechanisms. Accordingly, PKH26-labeled adipocyte-derived extracellular vesicles were counterstained with Hoechst (blue) before coincubation with 3T3-L1 cells. Confocal microscopy revealed that the 3T3-L1 cells effectively internalized the adipocyte-derived extracellular vesicles (Figure 2D).

### 3.3 Adipocyte-Derived Extracellular Vesicles from 3T3-L1 Cells Regulated by Nobiletin Inhibit Fat Accumulation

Adipose tissue, recognized as the largest organ for energy storage and secretion, has increasingly attracted scholarly interest regarding the secretion and functional roles of its exosomes.^43^ In this study, 3T3-L1 preadipocytes were cultured in 24-well plates, and during each medium change, differentiated preadipocytes, mature adipocytes, and adipocyte-derived exosomes regulated by nobiletin (10 μg/mL) were introduced concurrently (Figure 3A).

**Figure 3:**
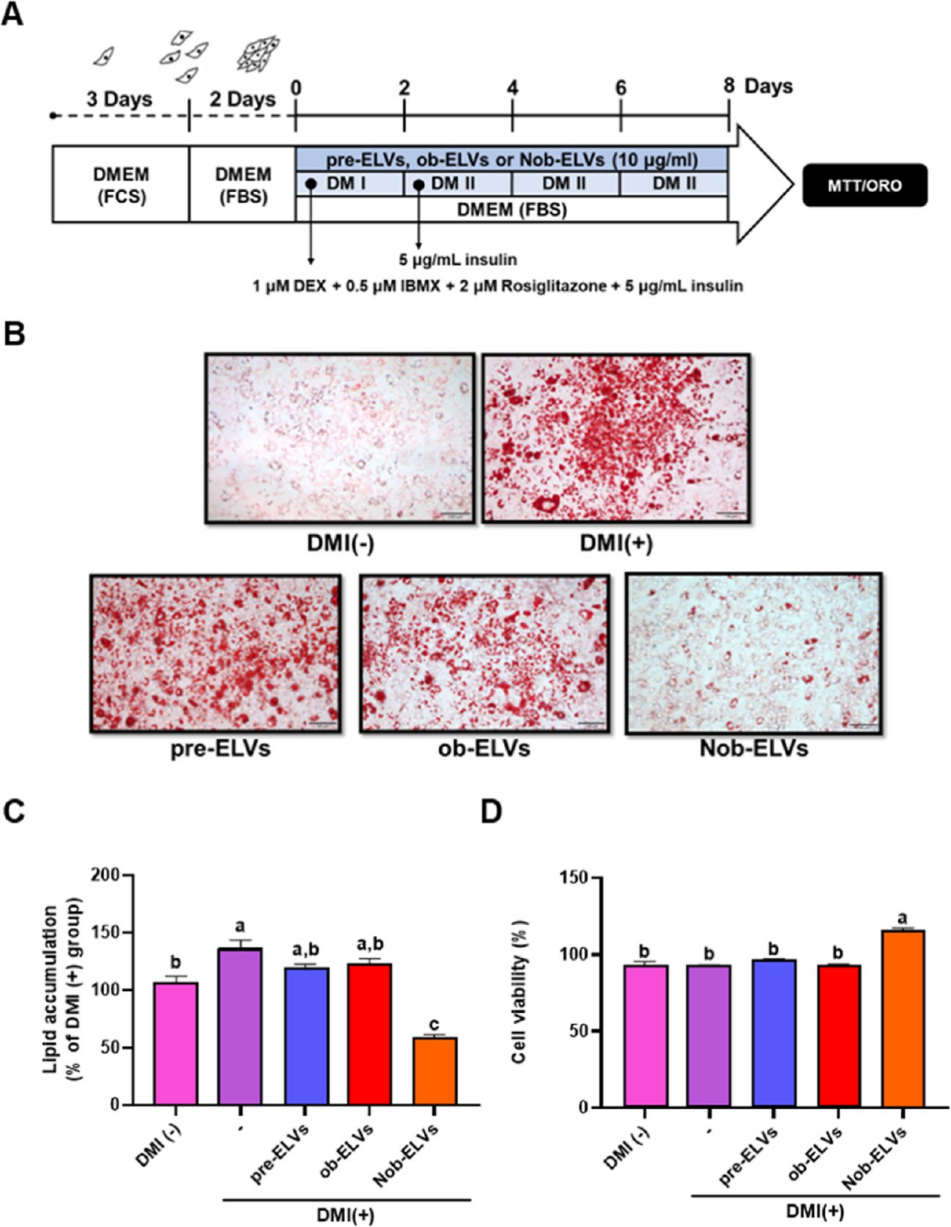
Nob-ELVs inhibit lipid accumulation in 3T3-L1 cells. (A) Diagram of adipocyte culture for the three groups of ELVs. (B) Representative photomicrographs of Oil Red O (ORO) stained 3T3-L1 adipocytes. (C) Effect of Nob-ELVs on lipid accumulation in 3T3-L1 adipocytes, determined using ORO staining. (D) Effect of Nob-ELVs on cell viability in 3T3-L1 adipocytes, determined using a 24 h MTT assay. A differentiation-inducing cocktail with or without Nob-ELVs was added to 3T3-L1 adipocytes for 8 days. Data are expressed as mean ± SEM (*n* = 3). Values with different letters are significantly different, *p* < 0.05.

This approach aimed to elucidate the effects of exosomes secreted by adipocytes in various physiological states on lipid metabolism. The experimental findings revealed a significant increase in the number of red oil droplets in the DMI­(+) group compared to the DMI(−) group. Notably, the pre-ELVs and ob-ELVs groups did not exhibit a reduction in the number of red oil droplets, remaining comparable to the DMI­(+) group. In contrast, the Nob-ELVs group demonstrated a significant decrease in both the area stained red and the number of oil droplets when compared to the DMI­(+) group, with cell morphology resembling that of the DMI(−) group, predominantly exhibiting a spindle shape (Figure 3B). Subsequently, oil red O was extracted from the cells using isopropanol for relative lipid quantification, and the quantitative results corroborated the microscopic observations, indicating that the lipid content in the DMI­(+) group was significantly elevated relative to the DMI(−) group. The pre-ELVs and ob-ELVs did not impede lipid accumulation, whereas the Nob-ELVs group exhibited a reduction of approximately 40% in intracellular lipid accumulation compared to the DMI­(+) group (Figure 3C).

After 48 h of treatment across the five experimental groups, cell viability remained above 90%, suggesting that Nob-ELVs did not adversely affect the viability of 3T3-L1 adipocytes and exhibited no cytotoxic effects (Figure 3D). The Nob-ELVs group effectively inhibited lipid accumulation within adipocytes, indicating that Nob-ELVs may mitigate intracellular lipid accumulation by either inhibiting the differentiation of 3T3-L1 preadipocytes or by reducing lipid production and synthesis, thereby demonstrating potential antiobesity properties.

### 3.4 Nobiletin Treatment Modulates miRNA Composition in Extracellular Vesicles

To investigate how extracellular vesicles from various sources regulate gene expression and influence cell function, this study employed RNA sequencing technology to analyze the differences in miRNA expression among the three distinct extracellular vesicles groups pre-ELVs, ob-ELVs, and Nob-ELVs. The Venn diagram illustrates 109 common miRNAs shared among the pre-ELVs, ob-ELVs, and Nob-ELVs groups, suggesting that these exosomes may all be involved in essential cellular processes. The pre-ELVs uniquely contain 390 miRNAs, indicating that extracellular vesicles derived from preadipose tissue may possess broader regulatory capabilities. The ob-ELVs uniquely contain 65 miRNAs, which may reflect specific regulatory functions associated with obesity-related extracellular vesicles. The Nob-ELVs uniquely contain 84 miRNAs, suggesting that nobiletin treatment may modify the miRNA composition of the extracellular vesicles, thereby impacting their function (Figure 4A).

**Figure 4:**
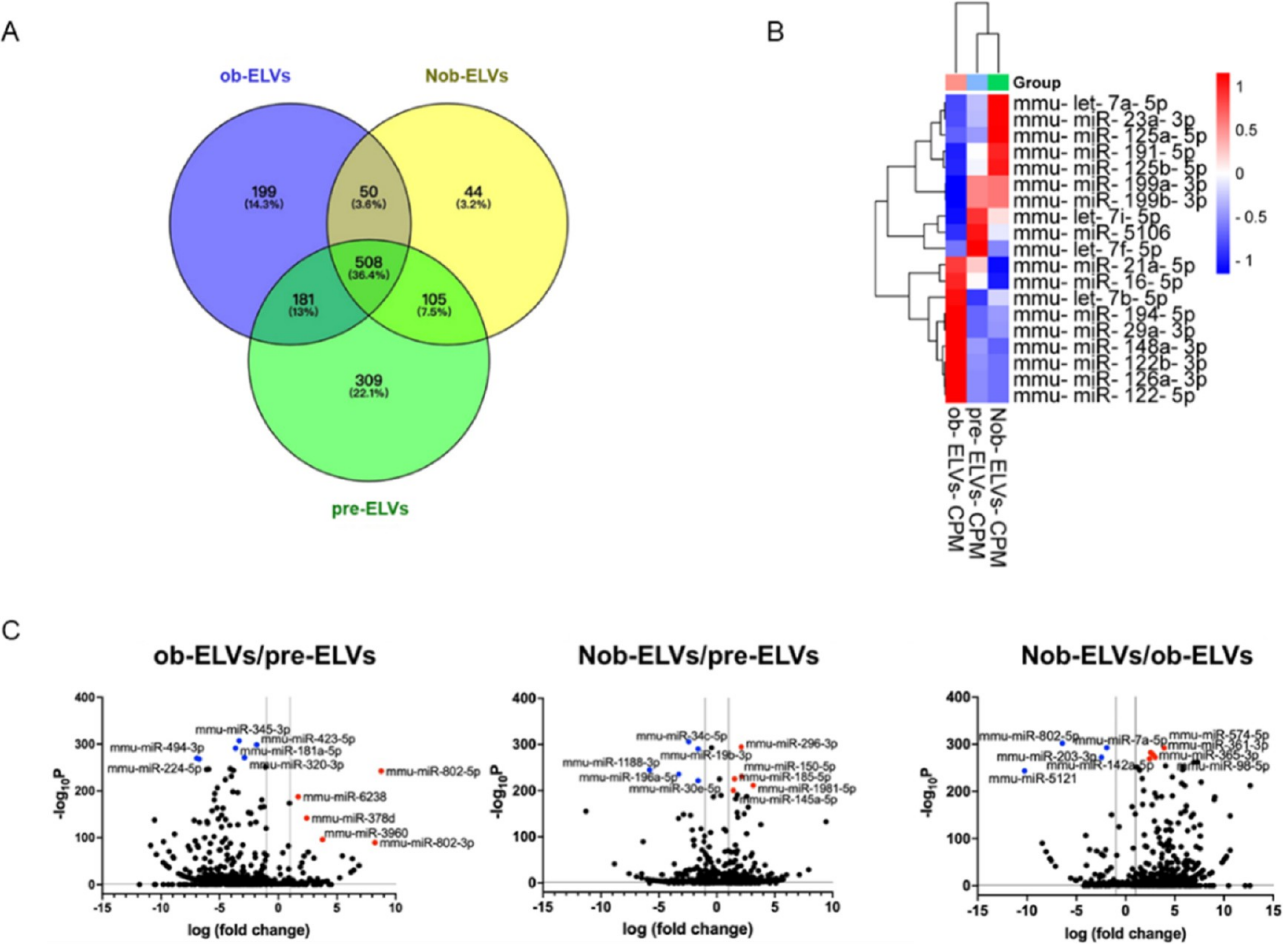
Nobiletin changes the expression of different miRNAs in exosomes secreted by 3T3-L1 adipocytes. (A) Venn diagram showing the distribution of miRNAs among different groups. (B) Heatmap of differentially expressed miRNAs among the groups. (C) Volcano plot displaying the fold change (log^2^ fold change) and statistical significance (*p*-value) of miRNA expression among the groups.

The heatmap illustrates the differential expression of miRNAs across the various groups. Significant differences in miRNA expression patterns are observed among the pre-ELVs, ob-ELVs, and Nob-ELVs groups. The color intensity indicates the levels of miRNA expression, with red representing high and blue denoting low expression. Notably, certain miRNAs are highly expressed in the ob-ELVs group while exhibiting low expression in the pre-ELVs group, which may be linked to obesity-related metabolic disorders. Additionally, other miRNAs display distinct expression patterns in the Nob-ELVs group, suggesting that nobiletin may influence biological activity by modulating miRNA expression. Furthermore, some miRNAs are highly expressed in the pre-ELVs group, potentially correlating with the normal physiological functions of preadipose tissue (Figure 4B).

The volcano plot illustrates fold change in miRNA expression and its statistical significance between the groups. Each point represents a miRNA, with the horizontal axis indicating the logarithm of the fold change (log^2^ fold change) and the vertical axis representing −log^10^ (*p*-value). Points located above the chart and far from the center line indicate miRNAs that are statistically significantly differentially expressed. Compared to ob-ELVs, miR-802-5p, miR-7a-5p, miR-203-3p, miR-142a-5p, and miR-5121 are significantly downregulated in Nob-ELVs. Conversely, miR-574-5p, miR-361-3p, miR-365-3p, and miR-98-5p are significantly upregulated in Nob-ELVs. These miRNAs are relatively more abundant in Nob-ELVs following nobiletin treatment, which may be associated with the protective effects of nobiletin, such as enhancing cellular antioxidant capacity or regulating lipid metabolism. In comparison to pre-ELVs, the microRNAs miR-34c-5p, miR-19b-3p, miR-1188-3p, miR-196a-5p, and miR-30e-5p exhibit significant downregulation in Nob-ELVs. Conversely, miR-296-3p, miR-150-5p, miR-185-5p, miR-1981-5p, and miR-145a-5p show significant upregulation in Nob-ELVs. The elevated levels of these microRNAs in exosomes following nobiletin treatment may be associated with the compound’s specific biological functions, which include the promotion of cellular repair mechanisms and the enhancement of insulin sensitivity. In comparison to pre-ELVs, the microRNAs miR-802-5p, miR-6238, miR-378d, miR-3960, and miR-802-3p exhibit significant upregulation in ob-ELVs. The elevated levels of these miRNAs in ob-ELVs may be indicative of metabolic disturbances or inflammatory responses associated with obesity. Notably, miR-802-5p is downregulated in both Nob-ELVs and ob-ELVs, suggesting that nobiletin may exert regulatory effects on this microRNA, potentially alleviating the adverse consequences of obesity. Conversely, the expression levels of miR-345-3p, miR-423-5p, miR-181a-5p, miR-320-3p, miR-494-3p, and miR-224-5p are significantly diminished in ob-ELVs when compared to pre-ELVs. These microRNAs may be relatively abundant in pre-ELVs but demonstrate a declining trend in ob-ELVs, implying that obesity may contribute to the downregulation of specific microRNAs that are crucial for maintaining normal physiological functions (Figure 4C).

### 3.5 Nob-ELVs Mitigate Weight Gain and Improve Food Conversion Efficiency in Mice on High-Fat Diets

From the photographs of the mice in each group, it is evident that the mice in the high-fat diet (HFD) group are significantly larger than those in the normal diet (ND) group, confirming that a high-fat diet leads to weight gain. In contrast, the body sizes of the mice in the HFD + pre-ELVs group, HFD + ob-ELVs group, and HFD + Nob-ELVs group fall between those of the ND group and the HFD group (Figure 5A).

**Figure 5:**
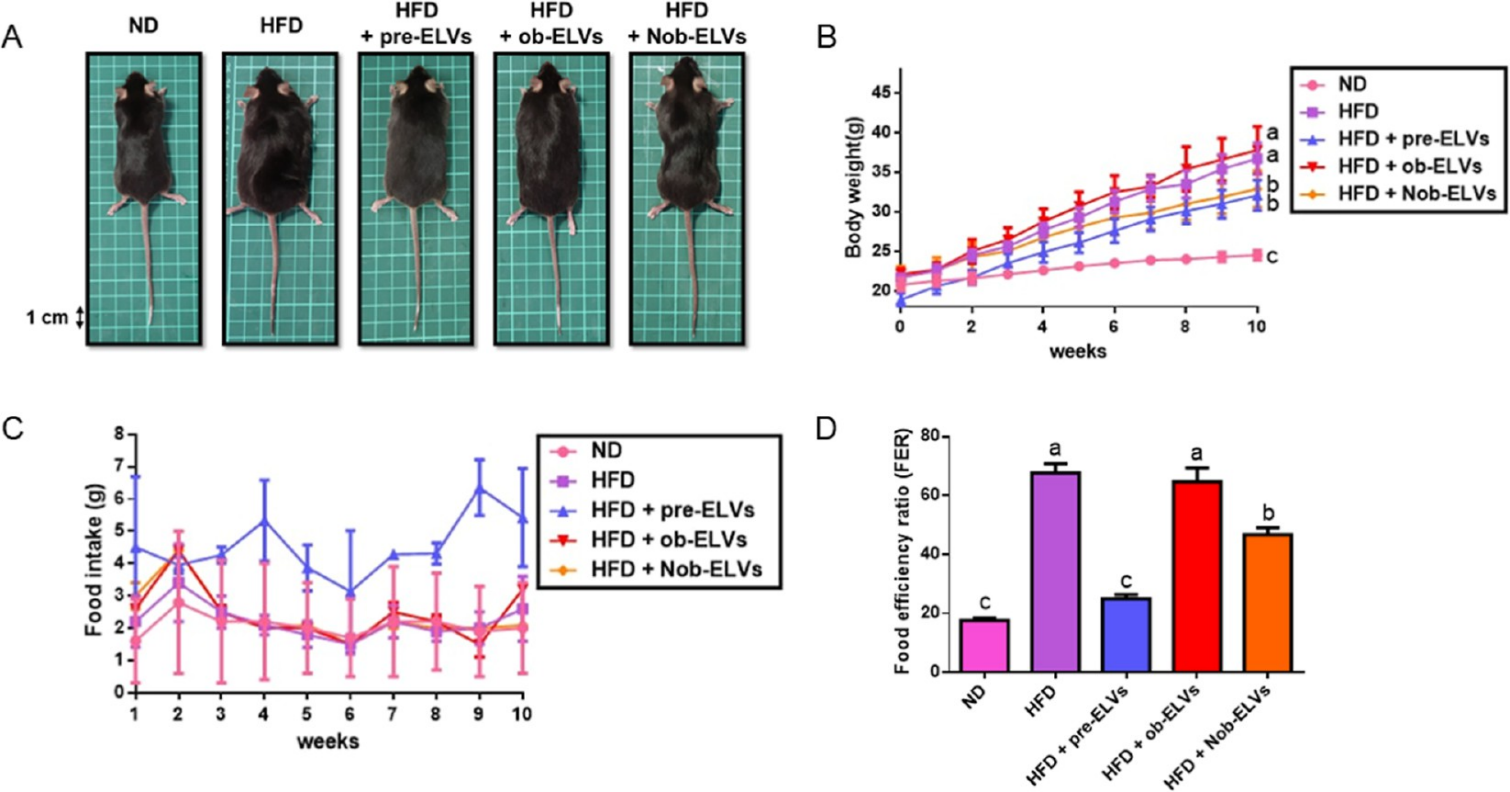
Mice fed HFD supplemented with adipocyte-derived extracellular vesicles for 10 weeks. (A) Representative photographs of each group of mice are shown at the end of week 10. (B) Body weight. (C) Food intake. (D) Food efficiency ratio (FER) was calculated with the equation: FER = (body weight gain (g)/food intake (g)) × 100. The average body weight of each group was expressed as mean ± SEM (*n* = 6 per group). The significant difference was analyzed by one-way ANOVA and Tukey’s range test. Values with different letters (a–c) are significantly different (*p* < 0.05) between each group.

Trends in weight change of each group of mice were monitored over a period of 10 weeks. The ND group exhibited the smallest weight gain, displaying a relatively flat ascending curve, while the HFD group experienced the most significant weight gain, characterized by the steepest slope of curve. The weight-gain trend of HFD + ob-ELVs closely mirrored that of the HFD group. In contrast, the weight gain of the mice in the HFD + Nob-ELVs group was significantly suppressed, demonstrating a marked reduction compared to the simple HFD group. This indicates that Nob-ELVs were most effective in mitigating weight gain induced by a high-fat diet (Figure 5B).

The food intake of mice in the ND group, HFD group, and HFD + ob-ELVs group remained relatively stable. In contrast, the food intake of the HFD + pre-ELVs group was slightly higher at certain time points compared to the other groups. Notably, the food intake of the HFD + Nob-ELVs treatment group was similar to that of the HFD group, with no significant difference observed between them. This indicates that the exosomes treated with nobiletin did not have a noticeable effect on the food intake of the mice (Figure 5C).

The food efficiency ratio (FER) of the ND group is the lowest among the groups studied. In contrast, the FER of the HFD group is significantly higher than that of the ND group. There is no significant difference in FER between the HFD + ob-ELVs and the HFD group. However, the FER of the HFD + pre-ELVs group is significantly lower than that of the HFD group. A key finding is that the FER value of the HFD + Nob-ELVs group is significantly lower than that of the HFD group and is comparable to that of the ND group (Figure 5D). These experimental results suggest that Nob-ELVs may inhibit weight gain in mice on a high-fat diet by influencing the efficiency of food conversion into body weight. This effect may be associated with alterations in the cargo of the exosomes, impacting lipid metabolism.

### 3.6 Nob-ELVs Ameliorate Blood Biochemical Abnormalities Induced by High-Fat Diet in a Murine Model

Obesity is associated with hyperlipidaemia, and excessive consumption of fatty acids can lead to inflammation and hepatic damage. Consequently, this study aims to investigate blood lipid profiles and liver function markers through the analysis of serum biochemical parameters. The findings indicate that a high-fat diet significantly influences biochemical metrics in a murine model, resulting in increased levels of the liver function enzyme aspartate aminotransferase (AST) and elevated blood lipid concentrations, including triglycerides (TG), total cholesterol (TC), high-density lipoprotein cholesterol (HDL-C), and low-density lipoprotein cholesterol (LDL-C). The research demonstrated that treatment with Nob-ELVs markedly ameliorated the biochemical disturbances induced by a high-fat diet, normalizing the affected parameters. In contrast, the administration of ob-ELVs did not yield significant improvements and, in certain instances, exacerbated specific metrics such as TG. Furthermore, pre-ELVs treatment resulted in partial amelioration of some indicators, including TG (Table 1). Notably, Nob-ELVs significantly rectified the biochemical abnormalities associated with a high-fat diet, restoring parameters such as TC, HDL-C, and LDL-C to baseline levels. These findings suggest that Nob-ELVs have the potential to enhance biochemical indicators related to lipid metabolism.

**Table 1:** Effects of Adipocyte-Derived Extracellular Vesicles on Serum Biochemical Parameters^a^

	ND	HFD	HFD + pre-ELVs	HFD + ob-ELVs	HFD + nob-ELVs
AST (U/L)	92.2 ± 45.9^a^	76.5 ± 37.3^ab^	82.3 ± 39.2^b^	56.0 ± 21.2^ab^	58.4 ± 19.4^b^
ALT (U/L)	18.9 ± 5.2^a^	15.0 ± 4.7^a^	12.2 ± 2.4^a^	13.3 ± 3.8^a^	9.2 ± 1.6^a^
TG (mg/dL)	34.0 ± 8.2^ac^	20.8 ± 3.2^bc^	45.9 ± 15.8^a^	25.3 ± 4.8^bc^	18.9 ± 4.2^b^
TC (mg/dL)	45.3 ± 4.8^c^	101.4 ± 7.3^a^	96.2 ± 7.9^a^	90.9 ± 10.2^a^	68.9 ± 12.3^b^
HDL-C (mg/dL)	37.1 ± 3.7^c^	77.9 ± 4.8^a^	76.7 ± 4.9^a^	69.1 ± 8.9^a^	54.1 ± 10.2^b^
LDL-C (mg/dL)	5.0 ± 1.2^d^	18.3 ± 3.2^ac^	13.9 ± 2.6^bc^	15.4 ± 1.4^c^	11.6 ± 2.1^b^

a Data are expressed as mean ± SEM (*n* = 6 per group). Significant differences were analyzed by one-way ANOVA and Tukey’s range test. Values with different letters (a–c) are significantly different (*p* < 0.05) between each group.

### 3.7 Nob-ELVs Reduce Fat Accumulation in Mice on a High-Fat Diet

Observational analysis revealed that the liver coloration in the high-fat diet (HFD) group and the HFD combined with ob-ELVs group exhibited a significantly more anemic appearance compared to the normal diet (ND) group. Conversely, the livers in the HFD combined with Nob-ELVs and HFD combined with pre-ELVs groups, while predominantly orange/brown, displayed a slightly more reddish hue than that observed in the HFD group. No significant differences were noted in the morphological characteristics of the kidneys and spleens across the various groups. Furthermore, the perigonadal fat, mesenteric fat, interscapular brown fat, beige fat, and perirenal fat regions in the HFD group were all significantly larger than those in the ND group. The fat volume in the HFD combined with ob-ELVs group closely resembled that of the HFD group, whereas the fat volumes in the HFD combined with Nob-ELVs and HFD combined with pre-ELVs groups were reduced in comparison to the HFD group. There were no significant differences in fat appearance between the HFD combined with ob-ELVs and HFD groups (Figure 6A).

**Figure 6:**
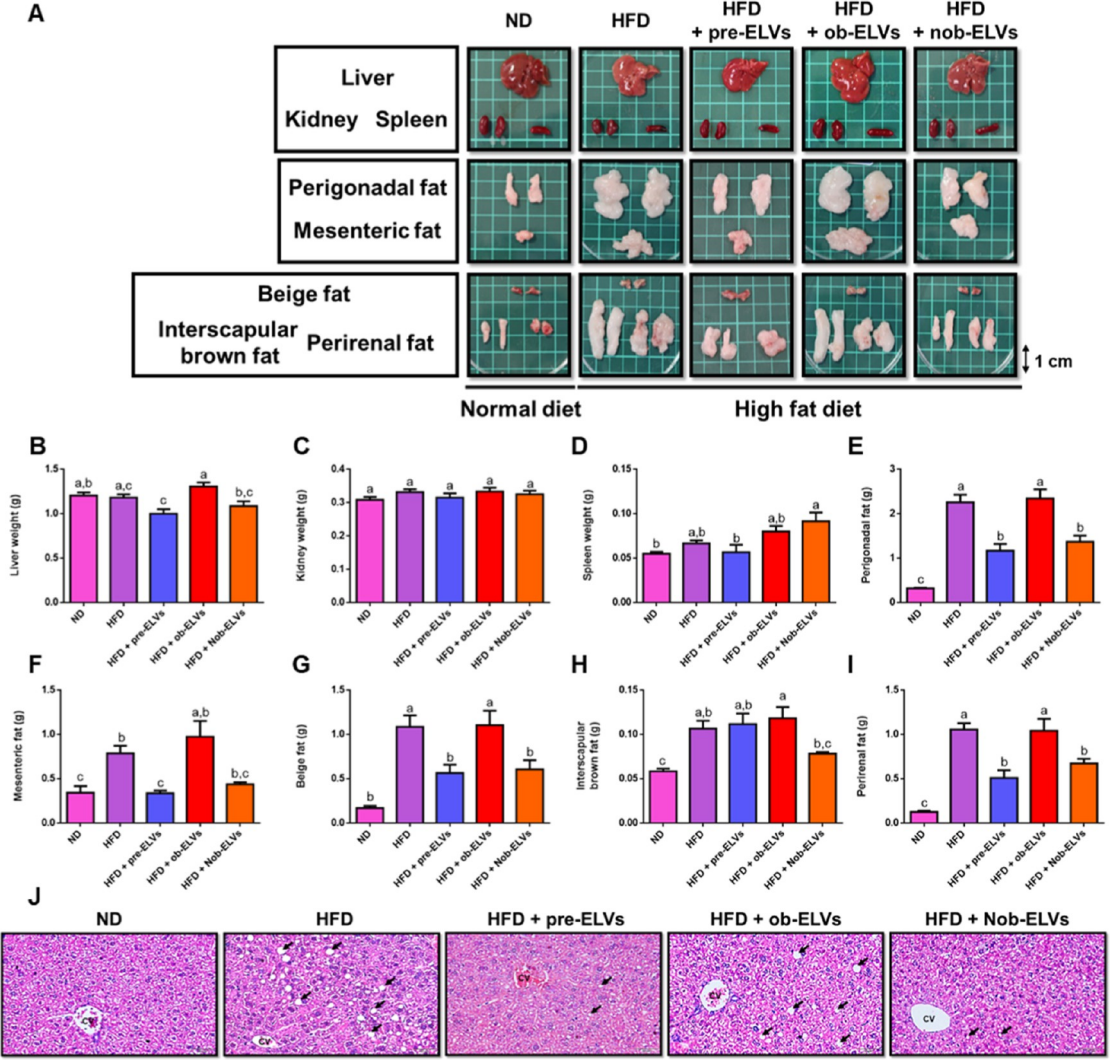
Impact of adipocyte-derived extracellular vesicles on the appearance and weight of organs in C57BL/6J mice subjected to a high-fat diet. Organs were excised and weighed immediately after sacrifice. (A) Representative photographs of each group. The weights of (B) liver, (C) kidney, (D) spleen, (E) perigonadal, (F) mesenteric, (G) beige, (H) interscapular brown, and (I) perirenal adipose tissues are presented. (J) Pathological assessment of the liver using H&E staining, measured at 200× magnification. Data are expressed as mean ± SEM (*n* = 6 per group). Significant differences were analyzed using one-way ANOVA followed by Tukey’s range test. Values with different letters (a–c) significantly different (*p* < 0.05) between each groups.

The analysis revealed no significant differences in the weights of the liver, kidneys, and spleen across the experimental groups (Figure 6B–D). However, the findings regarding visceral fat weight indicate that the total fat weight in the HFD group is significantly higher than that in the ND group. Furthermore, there are no notable differences in the weights of any type of visceral fat between the HFD + ob-ELVs and HFD groups (Figure 6E–I). In contrast, the weights of perigonadal fat, beige fat, and perirenal fat in the HFD + Nob-ELVs and HFD + pre-ELVs groups are significantly lower when compared to the HFD group (Figure 6E,G,I). These results suggest that Nob-ELVs are effective in reducing fat tissue accumulation in various regions of mice subjected to a high-fat diet.

Histological analysis of liver tissue sections revealed varying levels of lipid droplet accumulation among the different experimental groups. The liver tissue from the HFD group exhibited a significant increase in lipid droplet accumulation compared to the ND group, which is consistent with the established association between high-fat diets and the development of fatty liver disease. The liver tissue in the HFD + ob-ELVs group showed lipid droplet accumulation comparable to that of the HFD group. In contrast, the liver tissues from the HFD + pre-ELVs and HFD + Nob-ELVs groups demonstrated a trend toward reduced lipid droplet accumulation. Specifically, both the quantity and size of lipid droplets within hepatocytes were significantly decreased in these groups relative to the HFD group (Figure 6J). The Nob-ELVs appear to have the potential to ameliorate lipid droplet accumulation in the liver induced by a high-fat diet, possibly through the modulation of exosomal cargo that influences hepatic lipid metabolism.

### 3.8 Nob-ELVs Improve Lipid Oxidation and Metabolic Function in HFD-Induced Liver Disorders

This study investigates the effects of adipocyte-derived extracellular vesicles on liver lipid metabolism disorders induced by a high-fat diet, analyzing the expression changes of key proteins SIRT1, AMPK, PPARα, and FGF21, as well as how their upstream and downstream relationships regulate lipid oxidation (Figure 7A).

**Figure 7:**
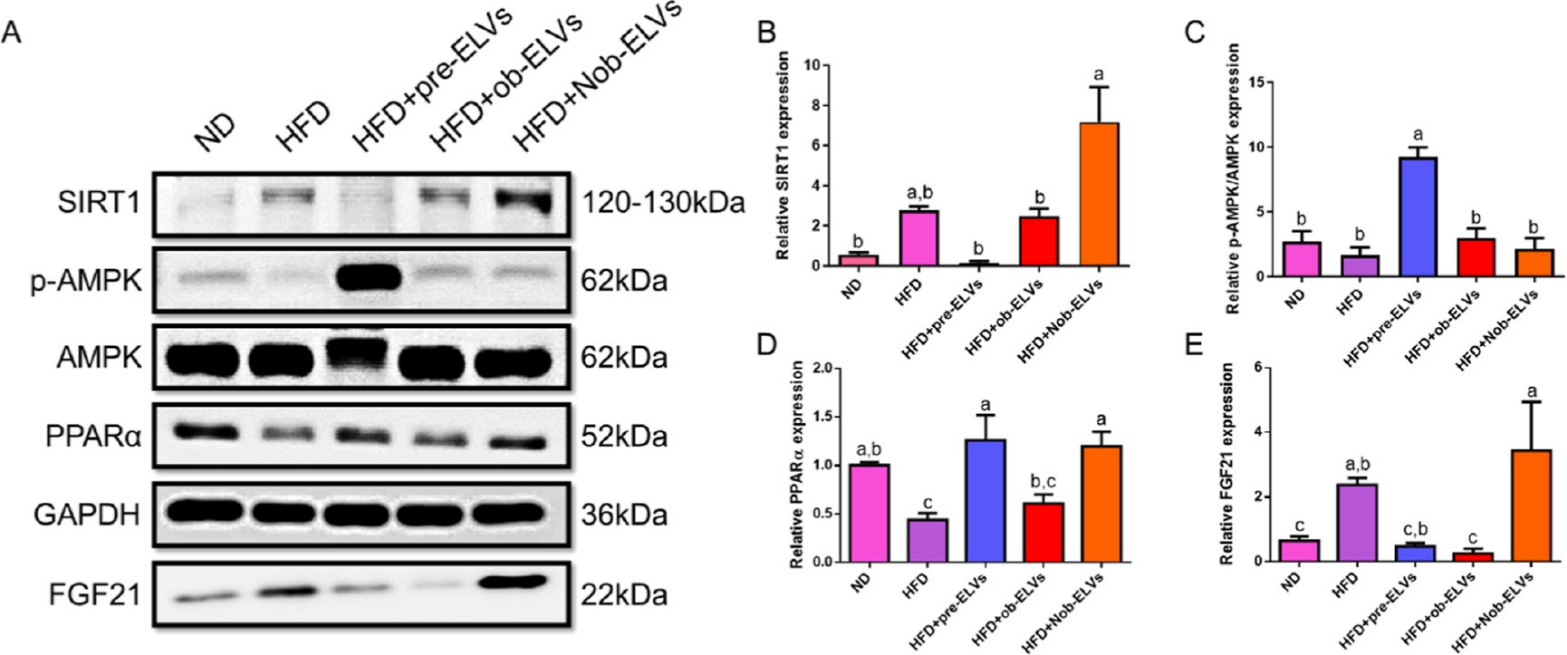
Nob-ELVs upregulate lipolysis markers in the liver of HFD-fed mice. Liver from HFD-fed mice administered with Nob-ELVs was analyzed for expression of lipolysis proteins. Relative expression of specific bands was quantified and presented as graphs. (A) Western blots of SIRT1, p-AMPK/AMPK, PPARα, and FGF21 proteins. Relative expression of (B) SIRT1 proteins, (C) p-AMPK/AMPK proteins, (D) PPARα proteins, and (E) FGF21 proteins. Data are expressed as mean ± SEM (*n* = 6 per group). Significant differences were analyzed by one way ANOVA and Tukey’s range test. Values with different letters (a–c) are significantly different (*p* < 0.05) between each group.

The expression level of the SIRT1 protein in the HFD group was significantly higher than that in the ND group, which may indicate a compensatory cellular response, combatting metabolic stress by increasing SIRT1 activity. However, the SIRT1 expression level in the HFD + pre-ELVs group was significantly lower than that in the HFD group, returning to levels comparable to those in the ND group. This suggests that pre-ELVs derived from normal adipose tissue can effectively regulate the overexpression of SIRT1 induced by a high-fat diet, normalizing its levels. In contrast, the SIRT1 expression level in the HFD + ob-ELVs group was similar to that in the HFD group, with no significant difference, indicating that ob-ELVs from obese adipose tissue are ineffective in regulating SIRT1. Notably, the SIRT1 expression level in the HFD + Nob-ELVs group exhibited an increasing trend compared to the HFD group, implying that nobiletin may further activate SIRT1 by modulating adipocyte-derived extracellular vesicles (Figure 7B).

The expression levels of AMPK protein in the HFD group exhibited a tendency to be lower compared to those in the ND group. This observation suggests that a high-fat diet may suppress AMPK activation, thereby diminishing fatty acid oxidation and promoting lipogenesis. However, AMPK expression level in the HFD + pre-ELVs group is significantly higher than that in the HFD group, suggesting that pre-ELVs can effectively activate AMPK. This activation may explain why pre-ELVs improve metabolic function. In contrast, AMPK expression levels in the HFD + ob-ELVs and the HFD + Nob-ELVs groups show a decreasing trend compared to the HFD group, indicating that both ob-ELVs and Nob-ELVs are ineffective in activating AMPK (Figure 7C).

The expression level of PPARα protein in the HFD group is significantly lower than that in the ND group, indicating that a high-fat diet suppresses PPARα expression, thereby affecting fatty acid oxidation. The PPARα expression level in the HFD + ob-ELVs group is comparable to that in the HFD group, showing no significant difference. Notably, the PPARα expression levels in the HFD + pre-ELVs and the HFD + Nob-ELVs groups are significantly higher than those in the HFD group, suggesting that both pre-ELVs and Nob-ELVs can effectively enhance PPARα expression (Figure 7D).

The expression level of FGF21 protein in the HFD group is significantly higher than that in the ND group. This increase may represent a compensatory response of the body to metabolic stress as it attempts to ameliorate metabolic disorders by elevating FGF21 levels. However, expression levels of FGF21 in the HFD + pre-ELVs and the HFD + ob-ELVs groups exhibit a decreasing trend compared to the HFD group, whereas the FGF21 protein expression level in the HFD + Nob-ELVs group shows an increasing trend relative to the HFD group. This finding suggests that nobiletin may enhance the expression of FGF21 by modulating exosomes, thereby improving metabolic function (Figure 7E).

### 3.9 Nob-ELVs Effectively Inhibit Fatty Acid Synthesis in HFD Mice

To investigate the effects of a high-fat diet on fatty acid synthesis, the expression changes of key proteins p-ACC/ACC, FASN, and CD36 were analyzed (Figure 8A). The expression level of ACC protein in the HFD group is significantly higher than that in the ND group, indicating that a high-fat diet promotes ACC expression, thereby increasing fatty acid synthesis. Notably, ACC expression in the HFD + pre-ELVs group is comparable to that in the HFD group, with no significant difference observed. This suggests that pre-ELVs derived from normal adipose tissue are ineffective in regulating the overexpression of ACC. In contrast, the ACC expression levels in the HFD + ob-ELVs and the HFD + Nob-ELVs groups are significantly lower than those in the HFD group, returning to levels similar to those in the ND group. This indicates that ob-ELVs and Nob-ELVs can effectively inhibit the overexpression of ACC, thereby reducing fatty acid synthesis (Figure 8B).

**Figure 8:**
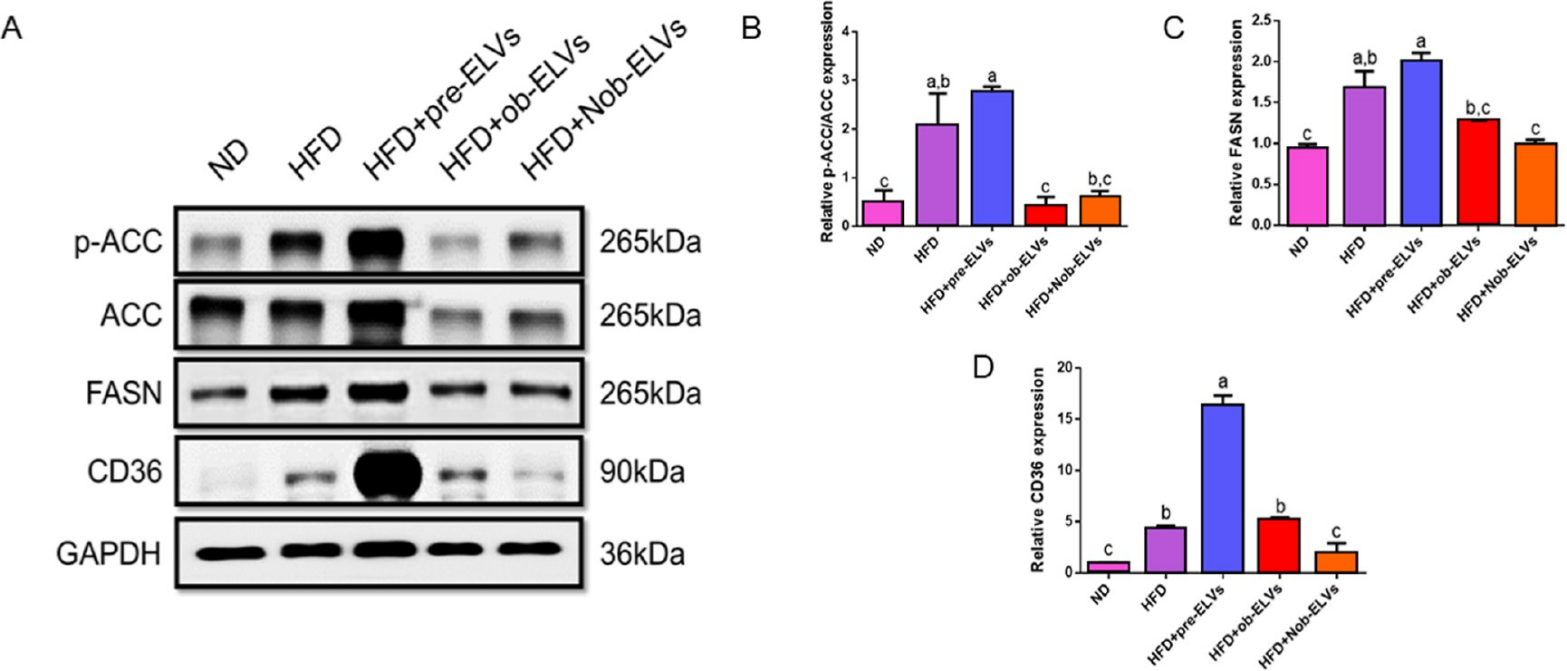
Nob-ELVs downregulate adipogenesis markers and lipid synthesis in the liver of HFD-fed mice. Liver from HFD-fed mice administered with Nob-ELVs was analyzed for expression of adipogenesis and lipid synthesis proteins. Relative expression of specific bands was quantified and presented as graphs. (A) Western blots of p-ACC/ACC, FASN, and CD36 proteins. Relative expression of (B) p-ACC/ACC proteins, (C) FASN proteins, and (D) CD36 proteins. Data are expressed as mean ± SEM (*n* = 6 per group). Significant differences were analyzed by one way ANOVA and Tukey’s range test. Values with different letters (a–c) are significantly different (*p* < 0.05) between each group.

The expression level of the FASN protein in the HFD group is significantly higher than that in the ND group, indicating that a high-fat diet promotes FASN expression, which in turn increases fatty acid synthesis. The FASN protein expression levels in the HFD + pre-ELVs and the HFD + ob-ELVs groups are comparable to those in the HFD group, with no significant differences observed. This suggests that both pre-ELVs and ob-ELVs are ineffective in regulating the overexpression of FASN. In contrast, the FASN expression level in the HFD + Nob-ELVs group is significantly lower than that in the HFD group, with levels similar to those in the ND group. This finding indicates that Nob-ELVs can effectively inhibit the overexpression of FASN, thereby reducing fatty acid synthesis (Figure 8C).

The expression level of the CD36 protein in the HFD group is significantly higher than that in the ND group, indicating that a high-fat diet promotes CD36 expression and increases fatty acid uptake. CD36 protein expression in the HFD + ob-ELVs group is comparable to that in the HFD group, with no significant difference observed. This suggests that ob-ELVs do not effectively regulate the overexpression of CD36. Interestingly, the CD36 expression level in the HFD + pre-ELVs group is significantly elevated compared to the HFD group, which may indicate a mechanism by which pre-ELVs enhance fatty acid uptake. Further research is necessary to confirm this. In contrast, the CD36 expression level in the HFD + Nob-ELVs group is significantly lower than that in the HFD group, with a level similar to that in the ND group. This finding indicates that Nob-ELVs can effectively inhibit the overexpression of CD36, thereby reducing fatty acid uptake (Figure 8D).

Based on these results, this study finds that pre-ELVs primarily exert their effects by regulating proteins associated with lipid oxidation, such as SIRT1, AMPK, PPARα, and FGF21. However, they do not significantly impact proteins related to lipid synthesis, such as ACC and FASN. The regulatory capacity of ob-ELVs on lipid synthesis-related proteins is also limited, as they can only reduce the expression of ACC. Notably, Nob-ELVs can effectively inhibit the overexpression of ACC, FASN, and CD36, suggesting that nobiletin may primarily reduce hepatic lipid accumulation by suppressing lipid synthesis.

## 4 Discussion

In the present investigation, we isolated exosomes released by 3T3-L1 adipocytes that were stimulated with nobiletin. This approach was undertaken to tackle the challenges associated with the low absorption rate and bioavailability of nobiletin, with the objective of evaluating their characteristics in the inhibition of lipid accumulation. Recent investigations have elucidated the mechanisms through which nobiletin inhibits lipid accumulation in adipocytes. While the current study demonstrates that a concentration of 100 μM nobiletin significantly influences lipid accumulation without cytotoxic effects, previous research indicates that a dosage of 120 μM not only enhances adiponectin secretion from 3T3-L1 adipocytes but also diminishes secretion of insulin resistance factor MCP-1.^44^ Consequently, to ensure safety, we opted for the highest concentration pertinent to lipid metabolism in 3T3-L1 adipocytes that does not induce cytotoxicity. The isolated Nob-ELVs were introduced into 3T3-L1 preadipocytes, and while they did not compromise cell viability, Nob-ELVs significantly inhibited lipid accumulation. This observation implies that Nob-ELVs may impede the differentiation of 3T3-L1 preadipocytes or the process of lipid synthesis, ultimately resulting in a decrease in intracellular fat accumulation. These results align with previous research indicating that nobiletin can inhibit lipogenesis through the modulation of cAMP/PKA/HSL and can suppress adipogenesis by influencing lipogenic transcription factors.^45^

Research has demonstrated that exosomes derived from adipocytes are associated with metastasis-associated lung adenocarcinoma transcript 1 (MALAT1), a long noncoding RNA that enhances the expression of mTOR in pro-opiomelanocortin (POMC) neurons located in the hypothalamus, while concurrently decreasing POMC expression. The results indicate that exosome-associated MALAT1 is significantly elevated in the adipocytes of obese mice and modulates mTOR signaling through the microRNAs miR-181b and miR-144.^46^ Additionally, exosomes from the adipocytes of obese mice were found to increase appetite and body weight in lean mice, whereas exosomes from lean mice resulted in a decrease in appetite and body weight in obese mice. Notably, the experiment did not reveal a significant increase in food intake and body weight in the ob-ELVs group, which may be attributed to variations in the exosomal content between mouse adipocytes and 3T3-L1 adipocytes. Furthermore, studies have indicated that, in the context of obesity, macrophages—specifically adipose tissue macrophages (ATMs)—infiltrate and accumulate within adipose tissue.^47^ ATMs serve as a significant source of pro-inflammatory cytokines, such as tumor necrosis factor-alpha (TNF-α) and interleukin-6 (IL-6), which disrupt insulin signaling pathways in adipocytes through the release of these cytokines.^48^ This disruption ultimately leads to insulin resistance, hyperglycaemia, and metabolic disturbances that contribute to the progression of obesity and type 2 diabetes. Consequently, the administration of exosomes from mature 3T3-L1 adipocytes did not result in an increase in body weight or appetite in the mice, and the underlying reasons for these discrepancies warrant further investigation.

Appetite regulation is a multifaceted physiological process influenced by a variety of factors, including the nervous system, hormonal signals, and metabolic status. The role of exosomes in appetite regulation represents only a small part of the broader regulatory framework, necessitating a comprehensive consideration of additional influencing factors. Furthermore, the HFD + Nob-ELVs group effectively reduced the food efficiency ratio (FER) and the final average body weight, suggesting that future research could investigate the miRNA-related pathways associated with Nob-ELVs to determine whether the antiobesity molecular mechanisms are connected to appetite regulation.

MicroRNA is a brief, single-stranded, noncoding RNA molecule that has the capacity to bind to the 3′ untranslated region (3′-UTR) of mRNA, thereby modulating gene expression through the inhibition of translation or the promotion of mRNA degradation. Evidence suggests that miRNAs are involved in the regulatory mechanisms of various biological processes related to obesity, including lipogenesis.^49^ The findings suggest that the composition of the miRNA transported by the exosomes is dependent on their source.^50^ The differential expression of specific miRNAs throughout the processes of adipogenesis, in mature adipocytes, and in the context of chronic obesity suggests their potential utility as biomarkers or therapeutic targets. Notably, miRNAs that exhibit upregulation in cases of persistent obesity are found to be downregulated during the adipogenic process. Conversely, certain miRNAs that are downregulated in individuals with obesity are upregulated in mature adipocytes. Furthermore, the induction of stress and hypertrophy in adipocytes results in the secretion of adipocyte-derived extracellular vesicles, which contain miRNA cargo molecules.^51^ In the present study, we examined the use of sequencing technology was investigate variations in miRNA expression in ob-ELVs, Nob-ELVs, and pre-ELVs. The volcano plot demonstrates that the three sources of adipocyte-derived extracellular vesicles influence the miRNA composition they harbor. Notably, miR-802-5p exhibits increased expression in ob-ELVs compared to both Nob-ELVs and pre-ELVs, suggesting its potential involvement in cellular functions associated with obesity. This observation is consistent with previous research indicating a correlation between miR-802-5p and T2DM.^52^ Studies have shown that the expression of miR-802-5p is elevated in adipocytes of obese individuals, where it has been found to disrupt insulin signaling pathways.^53^ Specifically, miR-802-5p derived from exosomes of hypertrophic adipocytes has the capacity to induce insulin resistance in cardiomyocytes, potentially through the targeting of heat shock protein 60 (HSP60).^54^ These exosomes, which are enriched in miR-802-5p, may propagate adverse effects in other tissues and organs, including cardiomyocytes, thereby contributing to systemic metabolic dysfunction. This suggests that ob-ELVs may facilitate intertissue transmission of detrimental effects via exosomal mechanisms, playing a significant role in the pathogenesis of obesity-related diseases, such as diabetes and cardiovascular disorders.^54^ Further investigation is warranted to elucidate the specific mechanisms involved and to assess their clinical implications.

Previous studies have demonstrated that in the livers of mice supplemented with nobiletin, the mRNA expression of genes involved in cholesterol synthesis and esterification (*Srebp2*, *Hmgcr*, *Acat2*), as well as lipogenesis (*Srebf1* and *Fas*), was significantly reduced. Conversely, the mRNA expression of *Pparα* in the liver was upregulated.^55^ In the present study, we conducted a more in-depth examination of the downstream molecular mechanisms through which Nob-ELVs exert their inhibitory effects on fat deposition. In the HFD group, the levels of SIRT1 protein are markedly elevated compared to those in the ND group, indicating a cellular adaptation to metabolic stress. Notably, the HFD + Nob-ELVs group exhibits a trend of increasing SIRT1 expression, suggesting that nobiletin may enhance SIRT1 activity through the modulation of exosomes derived from adipocytes. AMPK has the ability to suppress pathways associated with energy expenditure while concurrently promoting compensatory mechanisms aimed at re-establishing energy homeostasis.^56^ AMPK plays a crucial role in the activation of autophagy and the sirtuin protein SIRT1.^57^ Within hepatic tissue, AMPK enhances lipid accumulation by facilitating fatty acid oxidation through the activation of SIRT1, while simultaneously inhibiting metabolic processes such as lipid biosynthesis.^58^ Furthermore, the levels of PPARα are significantly diminished in the HFD group, which reflects a decrease in fatty acid oxidation and an increase in lipogenesis associated with high-fat dietary intake. Conversely, PPARα expression is significantly upregulated in the HFD + Nob-ELVs group, indicating a successful enhancement by nobiletin. Additionally, while FGF21 expression is elevated in the HFD group, likely as a compensatory response, it shows a further increase in the Nob-ELVs group, suggesting an improvement in metabolic function. Peroxisome proliferator-activated receptor alpha (PPARα) is a member of the PPAR protein family and functions as a transcription factor that plays a crucial role in regulating lipid metabolism.^59^ PPARα influences lipid metabolism by modulating the expression of its downstream target genes, which include fibroblast growth factor 21 (FGF21), carnitine palmitoyltransferase 1α (CPT1α), and acyl-CoA oxidase 1 (ACOX1).^60^ Activation of PPARα has been shown to lead to a reduction in plasma triglyceride levels, a decrease in obesity, and an improvement in hepatic steatosis. FGF21, a prominent member of the fibroblast growth factor family, is regulated by PPARα and is predominantly expressed in the liver, where it performs essential endocrine functions.^61^ Its primary roles include the regulation of lipolysis, the clearance of excess free fatty acids (FFA), and the enhancement of energy expenditure, all of which contribute to the negative regulation of hepatic or tissue steatosis and obesity.^62^ This suggests that exosomes derived from adipocytes treated with nobiletin may mitigate hepatic lipid accumulation by modulating the expression levels of hepatic PPARα and FGF21, thereby underscoring the significance of the liver-fat axis in lipid metabolism.

Moreover, the levels of lipogenic proteins, including ACC, FASN, and CD36, are significantly lower in the HFD + Nob-ELVs group compared to the HFD group. CD36 plays a crucial role in the uptake of long-chain fatty acids (LCFAs) within the liver.^63^ The enzyme long-chain acyl-CoA synthetase (ACSL) is essential for converting LCFAs into long-chain acyl-CoA, thereby facilitating their subsequent oxidation in the mitochondria. An increase in CD36 expression correlates with enhanced interaction between ACSL1 and CD36 in the mitochondria, promoting the transfer of additional LCFAs to ACSL1.^64^ This process augments the production of long-chain acyl-CoA, facilitating fatty acid oxidation and reducing fat accumulation in the liver. This suggests that, in contrast to pathways influenced by pre-ELVs, Nob-ELVs can effectively inhibit the overexpression of CD36, leading to a reduction in fatty acid uptake. The findings of this study indicate that pre-ELVs primarily exert their effects by modulating proteins associated with lipid oxidation, such as AMPK and PPARα, while having minimal impact on proteins involved in lipid synthesis, including ACC and FASN. In contrast, Nob-ELVs significantly enhance the expression of lipid oxidation-related proteins, such as SIRT1, PPARα, and FGF21, while concurrently inhibiting the overexpression of ACC, FASN, and CD36, thereby reducing fatty acid uptake and lipid synthesis. This differential regulation elucidates why pre-ELVs did not lead to improvements in blood lipid levels, whereas Nob-ELVs effectively decreased TC and LDL-C concentrations, resulting in a more pronounced overall improvement compared to pre-ELVs. Collectively, these results underscore the potential of nobiletin to regulate lipid metabolism in the mouse liver through the modulation of exosomes derived from adipocytes, thereby offering a novel approach for the development of antiobesity strategies aimed at inhibiting hepatic lipogenesis and reducing lipid accumulation.

In summary, the current investigation provides the inaugural evidence that exosomes induced by nobiletin and released from 3T3-L1 adipocytes alter the miRNA composition, leading to a decrease in lipid accumulation within adipocytes. In an experimental model utilizing obese mice, these exosomes demonstrated the capacity to regulate impaired lipid metabolism in the liver (Figure 9). These results indicate a potential therapeutic approach for the prevention of obesity and contribute to the growing body of knowledge regarding exosomes as an innovative mechanism in the fight against obesity. It is important to emphasize that while Nob-ELVs demonstrate the ability to inhibit fat accumulation and enhance lipid metabolism through the regulation of exosomal miRNA, *in vivo* investigations regarding their absorption and tissue distribution have yet to be conducted, indicating a need for further research. RNA sequencing has revealed significant alterations in the miRNA profile of Nob-ELVs, which affect various aspects of lipid metabolism. However, further experimental validation of the target genes associated with these miRNAs is essential. Future research should concentrate on elucidating the molecular mechanisms underlying specific miRNAs, including targeted gene regulation, signaling pathways, and intercellular communication, to clarify the causal relationships associated with metabolic enhancement. Comprehensive studies of these mechanisms will enhance our understanding of the pathogenesis of metabolic diseases and aid in the development of potential therapeutic interventions. Consequently, our findings provide a foundational basis for subsequent studies aimed at exploring the exosomal miRNA profile secreted by 3T3-L1 adipocytes treated with tangeretin, as well as assessing the molecular mechanisms underlying its antiobesity effects.

**Figure 9:**
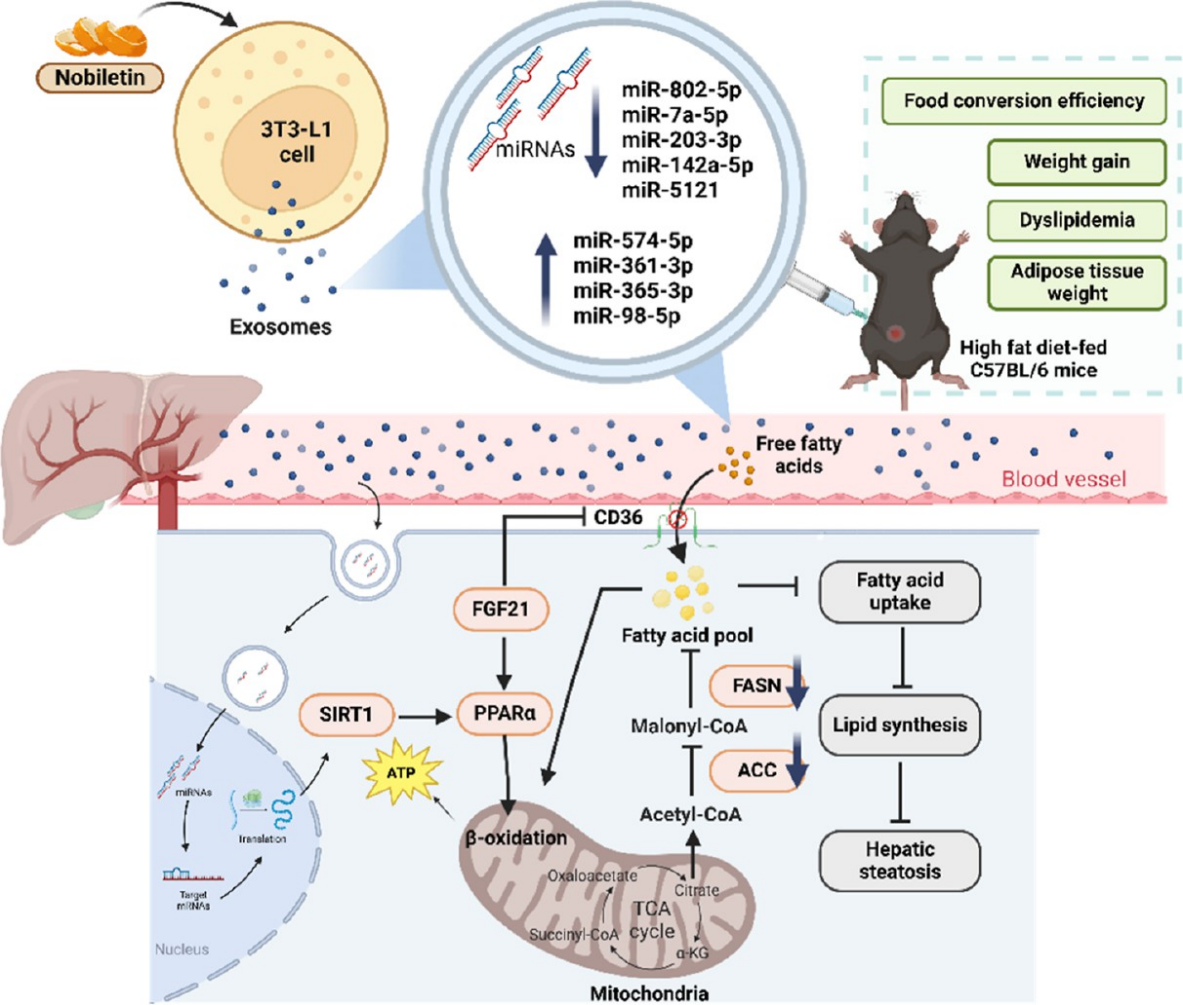
Nobiletin improves hepatic lipid metabolism in high-fat diet mice by modulating exosomal miRNAs.

## Abbreviations and Nomenclature

ACC: acetyl-CoA carboxylase
ALT: alanine aminotransferase
AMPK: AMP-activated protein kinase
AST: aspartate aminotransferase
AT: adipose tissue
CD36: cluster of differentiation 36
DAPI: 4′,6-diamidino-2-phenylindole
DEX: dexamethasone
DMEM: Dulbecco’s modified Eagle medium
DMI: differentiation medium
DMSO: dimethyl sulfoxide
ECL: enhanced chemiluminescence
ELISA: enzyme-linked immunosorbent assay
ELVs: extracellular vesicles
FASN: fatty acid synthase
FCS: fetal calf serum
FER: food efficiency ratio
FGF21: fibroblast growth factor 21
H&E: hematoxylin and eosin
HDL-C: high-density lipoprotein cholesterol
HFD: high-fat diet
Hmgcr: 3-hydroxy-3-methylglutaryl-CoA reductase
HPLC: high-performance liquid chromatography
HSP60: Heat shock protein 60
IBMX: 3-isobutyl-1-methylxanthine
LDL-C: low-density lipoprotein cholesterol
MCE: mixed cellulose esters
MCP-1: monocyte chemoattractant protein-1
miRNA: MicroRNA
mRNA: messenger RNA
MTT: thiazolyl blue tetrazolium bromide
MVB: multivesicular bodies
ND: normal diet
Nob-ELVs: nobiletin-treated exosomes
NTA: nanoparticle tracking analysis
ORO: Oil Red O
ob-ELVs: mature-adipocyte-derived extracellular vesicles
PBS: phosphate-buffered saline
PCR: polymerase chain reaction
PITA: probability of interaction by target accessibility
PMF: polymethoxyflavones
PPARα: peroxisome proliferator-activated receptor α
pre-ELVs: preadipocyte-derived extracellular vesicles
PVDF: polyvinylidene difluoride
RIPA: radioimmunoprecipitation assay
RNA: ribonucleic acid
RNase: ribonuclease
SEM: standard error of the mean
SIRT1: sirtuin 1
Srebp2: sterol regulatory element-binding protein 2
T2DM: type 2 diabetes mellitus
TC: total cholesterol
TEM: transmission electron microscopy
TG: triglycerides
TFF: tangential flow filtration
TPBS: Tween 20 in PBS
TSG101: tumor susceptibility gene 101
UTR: untranslated region
